# Tuning Nanofibrous Sensor Performance in Selective Detection of B-VOCs by MIP-NP Loading

**DOI:** 10.3390/nano15161220

**Published:** 2025-08-09

**Authors:** Antonella Macagnano, Fabricio Nicolas Molinari, Simone Serrecchia, Paolo Papa, Anna Rita Taddei, Fabrizio De Cesare

**Affiliations:** 1 Institute of Atmospheric Pollution Research (IIA), National Research Council (CNR), Montelibretti, 00010 Rome, Italy; fmolinari@unime.it (F.N.M.); simoneserrecchia@cnr.it (S.S.); paolo.papa@cnr.it (P.P.); decesare@unitus.it (F.D.C.); 2 Department of Chemical, Biological, Pharmaceutical and Environmental Sciences, University of Messina, 98166 Messina, Italy; 3 Electron Microscopy Section, High Equipment Centre, University of Tuscia, Building D, 01100 Viterbo, Italy; artaddei@unitus.it; 4 Department for Innovation in Biological, Agro-Food and Forest Systems (DIBAF), University of Tuscia, 01100 Viterbo, Italy

**Keywords:** MIP-nanoparticles, electrospun nanofibres, linalool sensing, B-VOC, humidity-tolerant sensor, MWCNT-PVP nanocomposites, selectivity, plant volatile monitoring, agriculture, environment

## Abstract

In this study, we investigate the effect of varying the loading of molecularly imprinted polymer nanoparticles (MIP-NPs) on the morphology and sensing performance of electrospun nanofibres for the selective detection of linalool, a representative plant-emitted monoterpene. The proposed strategy combines two synergistic technologies: molecular imprinting, to introduce chemical selectivity, and electrospinning, to generate high-surface-area nanofibrous sensing layers with tuneable architecture. Linalool-imprinted MIP-NPs were synthesized via precipitation polymerization using methacrylic acid (MAA) and ethylene glycol dimethacrylate (EGDMA), yielding spherical particles with an average diameter of ~135 nm. These were embedded at increasing concentrations into a polyvinylpyrrolidone (PVP) matrix containing multi-walled carbon nanotubes (MWCNTs) and processed into nanofibrous mats by electrospinning. Atomic force microscopy (AFM) revealed that MIP content modulates fibre roughness and network morphology. Electrical sensing tests performed under different relative humidity (RH) conditions showed that elevated humidity (up to 60% RH) improves response stability by enhancing ion-mediated charge transport. The formulation with the highest MIP-NP loading exhibited the best performance, with a detection limit of 8 ppb (±1) and 84% selectivity toward linalool over structurally related terpenes (α-pinene and R-(+)-limonene). These results demonstrate a versatile sensing approach in which performance can be precisely tuned by adjusting MIP content, enabling the development of humidity-tolerant, selective VOC sensors for environmental and plant-related applications.

## 1. Introduction

Monoterpenes emitted by plants serve as key molecular signals regulating physiological processes and mediating complex ecological interactions [1,2]. These biogenic volatile organic compounds (BVOCs) respond dynamically to developmental cues and environmental stressors, coordinating essential biological functions such as pollinator attraction, herbivore deterrence, and interplant communication.

Among them, limonene [3,4] and linalool [5,6,7,8,9,10,11,12] are particularly noteworthy, as their emission profiles are closely associated with specific phenological stages and stress-induced responses, thereby serving as reliable biomarkers for plant phenotyping and stress diagnostics.

Furthermore, monoterpenes such as α-pinene and linalool are major constituents of forest-emitted volatiles and have attracted growing interest for their therapeutic properties. In the context of forest bathing, a nature-based health practice, the inhalation of these compounds has been linked to psychological stress reduction and immune function enhancement [13,14].

Beyond their biological relevance, monoterpenes, which are classified as biogenic volatile organic compounds (BVOCs), also play a crucial role in atmospheric chemistry. Upon their release into the atmosphere, these compounds readily react with oxidants such as hydroxyl radicals, ozone, and nitrate radicals. Through these reactions, they contribute to the formation of tropospheric ozone (O_3_) and secondary organic aerosols (SOAs), thereby influencing both air quality and climate dynamics [15]. As a result, monoterpenes are increasingly considered in regulatory strategies aimed at mitigating both urban and rural air pollution. The real-time detection of monoterpenes is therefore essential both for gaining ecological insights and for supporting environmental risk assessment.

Despite their importance, the selective detection of these VOCs remains a challenge due to their structural resemblance, low concentrations, and high chemical reactivity. While conventional methods such as gas chromatography–mass spectrometry (GC-MS) and proton-transfer-reaction mass spectrometry (PTR-MS) [16,17] offer excellent sensitivity and selectivity, they rely on complex, costly, and non-portable equipment, limiting their applicability for on-site and real-time monitoring. These limitations have focused research on more compact, low-cost chemical sensors capable of discriminating among structurally similar VOCs under variable environmental conditions.

In recent years, researchers have developed multiple sensor architectures using nanostructured molecularly imprinted polymers (MIPs) for selective VOC detection, each designed to enhance recognition efficiency and signal transduction. MIPs are synthetic receptors engineered with binding sites complementary in shape, size, and functional group orientation to a target analyte, mimicking the molecular recognition properties of natural antibodies or enzymes. Their high chemical stability, reusability, and design flexibility make them particularly attractive for integration into sensing platforms aimed at volatile, low-concentration, and chemically similar compounds such as monoterpenes [18,19,20,21]. The choice of MIP integration strategy plays a pivotal role in determining sensor performance, influencing key parameters such as sensitivity, selectivity, response time, and operational stability. Among the wide range of available approaches, several have been explored for VOC detection, each offering distinct advantages and limitations.

A fundamental distinction can be made between template imprinting, the conventional approach in which polymerization occurs around the target molecule, and porogen imprinting, where the template also acts as the solvent or is used in excess. The latter has proven especially effective in enhancing analyte accessibility in gas-phase sensing, facilitating faster diffusion and improved sensitivity in porous matrices [22].

In terms of integration, ex situ immobilization strategies—such as the drop-casting or spin-coating of pre-synthesized MIP nanoparticles—are among the most widely used due to their simplicity and compatibility with various substrates [23,24]. The thickness and homogeneity of the sensing layer can be tuned by adjusting the solution parameters; however, this approach often suffers from limited reproducibility, poor adhesion, and non-uniform particle dispersion. These limitations have led to the use of binding additives or adhesive interlayers, although such modifications can reduce the accessibility of recognition sites [25]. Covalent bonding and electrostatic assembly represent more robust alternatives. While covalent attachment ensures stable MIP anchoring via surface functionalization, it requires complex pretreatment steps. Electrostatic assembly, based on ionic or dipole–dipole interactions, offers a milder route with easier reversibility, though it may yield weaker interfacial stability over time [26].

To overcome limitations in adhesion and electrical contact, electropolymerization has emerged as a powerful technique, enabling the in situ formation of the MIP layer directly on the transducer surface in the presence of the template. This method enhances electron transfer efficiency, particularly in electrochemical sensors [27,28,29], although it may constrain control over porosity and hinder analyte diffusion due to the dense polymer network.

Alternatively, sol–gel embedding allows MIPs to be incorporated into hybrid inorganic–organic matrices with high mechanical and chemical resistance. However, dense gel structures can impede analyte transport and reduce sensitivity [30].

Layer-by-layer (LbL) assembly provides molecular-level control over MIP film architecture by the sequential deposition of MIP and polyelectrolyte layers. While this technique offers excellent structural precision, it is labour-intensive and not easily scalable [31].

Surface imprinting techniques based on polymer grafting directly from the transducer substrate provide excellent adhesion and thin film control, improving mass transport and response speed [32]. Nevertheless, the fabrication process requires precise optimization to avoid compromising film permeability.

Recent innovations have expanded toward microfluidic and 3D-printed platforms, where MIP-based sensors benefit from miniaturization, portability, and multiplexed detection, though these systems are still in the developmental stage [33,34].

Hybrid strategies have also emerged, combining MIPs with conductive nanomaterials such as carbon nanotubes (CNTs) or graphene to improve electronic conductivity and mechanical strength. Despite promising enhancements in sensitivity and stability, challenges remain in achieving homogeneous nanoparticle dispersion without phase segregation or aggregation [35]. These systems provide improved signal transduction and structural stability, yet they still face challenges in achieving homogeneous dispersion, often leading to phase separation or aggregation.

Among these nanostructured strategies, electrospinning stands out for its ability to create highly porous, high-surface-area fibrous matrices capable of hosting MIPs in 3D configurations. This technique allows for the fine-tuning of fibre morphology, mechanical robustness, and analyte diffusion pathways, making it particularly suitable for chemiresistive sensing platforms operating in the gas phase [36]. In our previous work [37], more specifically, we demonstrated that a sensor composed of electrospun PVP/MWCNT nanofibres embedding limonene-specific MIP-NPs, produced via template-assisted precipitation polymerization [38], exhibited excellent sensitivity and selectivity. In this approach, pre-synthesized MIP nanoparticles can be dispersed into a polymer solution and electrospun into non-woven fibre mats with high surface area and interconnected porosity. However, a key limitation of this configuration can be the poorly controlled spatial distribution of the MIP nanoparticles within the fibrous network, which can lead to heterogeneous sensing behaviour and reduced reproducibility. In the present study, we address this issue by systematically varying the MIP-NP loading and assessing its influence on the structural and functional properties of the sensor. This strategy enables improved control over MIP dispersion, ultimately contributing to a more reproducible, scalable, and tuneable sensing architecture for VOC detection.

To further assess the sensor’s versatility and robustness, we extend its application to a new molecular target: linalool, a monoterpene structurally related to limonene but characterized by distinct physicochemical properties. Compared to limonene, linalool contains a hydroxyl group that increases polarity, molecular weight (154.25 g mol^−1^ vs. 136.23 g mol^−1^), water solubility (~1.6 g L^−1^), and conformational flexibility. These differences are expected to impact adsorption kinetics, diffusion through the fibrous network, and interaction with the MIP recognition sites, making linalool a more demanding, yet ideal, benchmark for validating the sensing architecture. In addition to its structural characteristics, linalool plays a distinct physiological role in plants. While limonene is typically associated with responses to heat [39,40], wounding or herbivore attack [41,42,43], linalool is more often linked to reproductive and defence-related processes, such as flowering, fruit ripening, and pathogen infection.

Elevated linalool emissions have been reported during flower development in *Arabidopsis* [44], fungal infection in *Vitis vinifera* [45], and insect oviposition in Nicotiana species [46], underscoring its diagnostic value in plant stress phenotyping and its complementarity to limonene. In this work, we introduce a tuneable chemiresistive sensor architecture that, for the first time, systematically correlates MIP-NP loading with both structural and functional properties of electrospun nanofibres. This strategy enables precise control over sensitivity, selectivity, and dynamic response, offering a more reproducible and scalable approach compared to conventional MIP coatings. By extending this concept to structurally diverse targets such as linalool, we demonstrate how a single sensor architecture can be customized for the selective detection of different BVOCs, highlighting the adaptability and transferability of the proposed design. The present study focuses on a single, integrated sensor design, in which pre-synthesized MIP nanoparticles are embedded within a nanostructured scaffold of electrospun PVP and MWCNTs. The dual objective is to (i) investigate how the density of molecular recognition elements (MIP loading) influences the morphological, electrical, and sensing properties of the nanofibrous network and (ii) validate its applicability to a new molecular target, linalool, thereby demonstrating a strategy for tuning selectivity and performance within the same structural framework. This dual investigation reveals how optimizing the density of recognition elements affects sensor performance and supports the broader adaptability of the sensing strategy to other VOC targets. By simply replacing the MIP component, the same electrospun architecture can be customized for the real-time, selective detection of various biogenic volatiles, making it a promising approach for next-generation environmental and agricultural sensing technologies.

## 2. Materials and Methods

### 2.1. Materials

Polyvinylpyrrolidone (PVP; Mw 1,300,000 and 30,000), methacrylic acid (MAA; Mw 86.09), 2,2′-azobis(2-methylpropionitrile) (AIBN; Mw 164.21), ethylene glycol dimethacrylate (EGDMA; Mw 198.22), and multiwalled carbon nanotubes (MWCNTs; >99% carbon basis, outer diameter × length: 6–13 nm × 2.5–20 μm) were used as received, without further purification. The volatile organic compounds employed as analytes, linalool (LIN, 97%, Mw 154.25), R(+)-limonene (R-LIM, 96%, Mw 136.23), and α-pinene (α-PIN, 98%, Mw 136.23), were also used as received. To minimize oxidative degradation or volatilization, VOCs were stored in sealed amber vials under refrigeration (8 °C) and handled in a fume hood during experimental procedures. Solvents included absolute ethanol (EtOH; ≥99.8%, ACS reagent, Mw 46.07), acetonitrile (MeCN; ≥99.5%, ACS reagent, Mw 41.05), and *N*,*N*-dimethylformamide (DMF; 99.8%, ACS reagent, Mw 73.09). All chemicals were purchased from Merck KGaA (Darmstadt, Germany).

### 2.2. Molecularly Imprinted Nanoparticle (MIPNP) Synthesis

Molecularly imprinted nanoparticles (MIP-NPs) were synthesized via precipitation polymerization using linalool (LIN) as the template molecule, following the procedure previously described by Molinari et al. (2025), with slight modifications [37]. The pre-polymerization solution was prepared by dissolving 0.5 mmol of LIN, 2 mmol of methacrylic acid (MAA), and 4 mmol of ethylene glycol dimethacrylate (EGDMA) (molar ratio 1:4:8) in 50 mL of acetonitrile, which served as the porogenic solvent. The mixture was stirred for 5 min, sonicated at 15% amplitude for 20 min to promote template–monomer interactions, and stirred again for 20 min to allow thermal equilibration. After cooling below 25 °C, azobisisobutyronitrile (AIBN, 2 wt% of the total mixture) was added as the radical initiator. The solution was purged with nitrogen for 15 min and then polymerized at 60 °C for 6 h under a nitrogen atmosphere. The reaction mixture turned milky white, indicating nanoparticle formation. After cooling, the dispersion was stored at 5 °C overnight to promote sedimentation. The resulting MIP-NPs were recovered by decanting the supernatant and purified by four consecutive washing cycles with ethanol, followed by centrifugation at 5500 rpm for 10 min. GC–MS analysis of the supernatants (Trace 1310 GC/TSQ 8000 Evo, Thermo Fisher Scientific, Waltham, MA, USA) confirmed that more than 95% of the template was removed after the first two washing steps. Non-imprinted polymer nanoparticles (NIP-NPs), synthesized in the absence of the template molecule, had already been prepared and tested in our previous work. When exposed to various terpenes, including linalool, they exhibited only minor current fluctuations without any evidence of selective response [37]. For this reason, NIP-NPs were not included in the present study.

### 2.3. MWCNT Dispersion

A stable dispersion of multi-walled carbon nanotubes (MWCNTs) at 0.7 wt% in *N*,*N*-dimethylformamide (DMF) was prepared by alternating cycles of probe sonication (30% amplitude) (VCX 500 Vibra-Cell ultrasonic processor, Sonics & Materials, Inc., Newtown, CT, USA) and magnetic stirring. To suppress aggregation and enhance dispersion stability, polyvinylpyrrolidone (PVP K30) was added as a stabilizing agent. This procedure was adapted and optimized from previously reported protocols [21,37,47].

### 2.4. Electrospun Layer Fabrication

Electrospinning solutions with increasing MIP-NP loadings were prepared by dispersing 10, 105, and 180 mg of MIP-NPs, respectively, in 1 mL of the pre-sonicated MWCNT dispersion in DMF. Each suspension was then probe-sonicated for 25 min at 10% amplitude to ensure uniform nanoparticle dispersion. Subsequently, 4 mL of a 12 wt% PVP solution in ethanol was added under magnetic stirring, followed by an additional 25 min of sonication in an ultrasonic bath (Branson M1800, Branson Ultrasonics, Danbury, CT, USA), equipped with a 40 kHz sweep-frequency transducer. The final MIP-to-PVP mass ratios in the electrospinning solutions were approximately 2.1%, 23%, and 40%, respectively. Electrospinning was carried out using a Fluidnatek^®^ LE-50 system (Bioinicia, Paterna, VAL, Spain). The process parameters were empirically optimized to achieve a stable and continuous jet, producing uniform fibres with minimal diameter variation and complete solvent evaporation before deposition. The needle-to-collector distance was set at 14 cm, and electrospinning was performed under ambient conditions (RH ~35%, T ~20 °C) with a fixed solution feed rate of 200 µL/h for all formulations. A dual-voltage configuration (+8 kV at the needle and −2 kV at the grounded collector) was applied to ensure stable Taylor cone formation and continuous fibre deposition for the selected solution conductivity. Electrospun fibres were deposited directly onto the sensor transducer surface, which was secured on the collector using conductive metallic tape to ensure stable electrical contact. Each deposition cycle lasted approximately 3 min, resulting in the formation of a continuous, homogeneous nanofibrous mat across the interdigitated microelectrode area.

### 2.5. UV-Crosslinking Process

The MIP-loaded nanofibres were irradiated with a 500 W broad-spectrum UV lamp (Polymer Helios, Italquartz, Cambiago, MI, Italy) for 10 min to promote photo-crosslinking within the PVP matrix. This post-treatment step is crucial because unmodified PVP is highly water-soluble and undergoes severe structural deformation when exposed to high humidity. UV irradiation generates free radicals that promote covalent crosslinking between polymer chains, thereby making the nanofibrous network insoluble, structurally stable, and resistant to humidity-induced swelling [48].

### 2.6. Interdigitated Microelectrode Layout

Interdigitated microelectrode (IDE) chips (Micrux Technologies, Gijón, Spain) were employed as transducing elements. Each device comprised a borosilicate glass substrate (10 × 6 × 0.75 mm) patterned with 120 pairs of platinum/titanium electrodes. The metallic lines were 10 μm wide, 3.5 mm long, and 150 nm thick, with an inter-electrode spacing of 10 μm. Before functionalization, the IDE chips underwent a standardized multistep cleaning procedure to ensure optimal surface conditions. Initially, the devices were rinsed with a mild laboratory detergent solution to remove organic contaminants. This was followed by immersion for 60 min in a “base piranha” solution (NH_4_OH:H_2_O_2_, 3:1 *v*/*v*) at 60 °C in a fume hood to promote thorough surface activation. Subsequently, the chips were extensively rinsed with Milli-Q water (resistivity ≈ 18 MΩ·cm), flushed with isopropyl alcohol, and dried under a gentle stream of nitrogen gas.

### 2.7. Scanning Electron Microscopy (SEM)

The morphology of the MIP-NPs was characterized by scanning electron microscopy (SEM, JEOL JSM-6010LA; JEOL Ltd., Tokyo, Japan) operating in secondary electron imaging mode (SEI) at an accelerating voltage of 5 kV. Samples were mounted on aluminium stubs using conductive carbon tape and sputter-coated with a 2 nm gold layer (Quorum Q150R ES, Quorum Technologies, East Sussex, UK) to improve surface conductivity and image quality. Nanoparticle size measurements were performed on SEM micrographs using the open-source software ImageJ, equipped with the DiameterJ (version 1.018) plugin, based on 100 individual particle measurements.

### 2.8. Atomic Force Microscopy (AFM)

The surface topography of the nanostructured layers was characterized by atomic force microscopy (AFM) using a Nanosurf Flex-AFM system (version 5-C3000, Nanosurf AG, Liestal, Switzerland). Measurements were conducted in continuous mode with aluminium-coated silicon tips (DynAl90), featuring a resonance frequency of approximately 190 kHz and a nominal spring constant of 18 N/m. Scans were collected over multiple areas (60 × 60 µm, 20 × 20 µm, and 10 × 10 µm) to assess both microscale and nanoscale features. The resulting topographic data were processed and analysed using a free open-source platform (Gwyddion software, version 2.64).

### 2.9. Electrical and Sensing Measurements

Sensing devices based on MIP-loaded nanofibres deposited onto interdigitated electrodes (MIP-NFs/IDEs) were integrated into a sealed test chamber with an internal volume of approximately 1 mL. Electrical characterization was performed using a Keithley 6517 electrometer (Keithley Instruments, Solon, OH, USA), which provided both the bias voltage and the current measurements. Data acquisition was managed via LabVIEW 2014 software (National Instruments, Austin, TX, USA) installed on a dedicated PC. Baseline measurements were carried out under a flow of clean, dry air (purity 5.0, Nippon Gases Europe, Madrid, Spain) at ambient temperature and controlled relative humidity. For static I–V characterization, the voltage was swept between −4.0 V and +4.0 V, and the electrical resistance (R) of the nanofibrous sensing layer was calculated from the linear region of the I–V curve using Ohm’s law. Dynamic gas sensing tests were conducted by applying a constant bias of +3 V across the IDEs at a stable temperature of 25 °C. Gas flow control was achieved using an MKS 247 multi-channel unit connected to four mass flow controllers (MFCs), each capable of regulating flows from 0 to 200 standard cubic centimetres per minute (sccm). Humidity modulation was achieved by directing dry air through a water-filled bubbler; relative humidity and temperature were continuously monitored using an HIH 4602 sensor (Honeywell International Inc., Charlotte, NC, USA). Target analytes, including linalool (LIN), R-(+)-limonene (R-LIM), and α-pinene (α-PIN), were introduced by bubbling part of the carrier gas through liquid VOC standards to generate saturated vapours. They were subsequently mixed with the main gas stream in a 50 mL pre-chamber (mixing chamber) to ensure homogeneous analyte distribution before reaching the sensor. Each exposure cycle was initiated only after complete baseline current recovery under clean air. The sensor response was expressed as the relative current variation, ΔI/I_0_, where ΔI is the difference between the analyte-induced current and the baseline current (I_0_), corresponding to the steady-state signal under clean air.

## 3. Results and Discussion

### 3.1. MIP-NP Characterization

**Figure 1:**
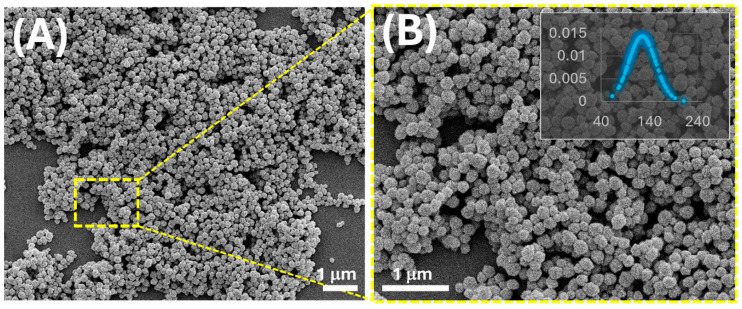
SEM images (secondary electrons, 5 kV) of linalool-imprinted polymer nanoparticles (MIP-NPs) obtained by precipitation polymerization, shown at two different magnifications: (**A**) overview of the nanoparticle distribution; (**B**) magnified view highlighting the spherical-to-faceted morphology and moderate surface roughness. The inset in panel (**B**) reports the normalized size distribution of particle diameters, with an average diameter of 135 ± 28 nm (mean ± standard deviation, *n* = 100).

The morphology and size distribution of linalool-targeted molecularly imprinted polymer nanoparticles (MIP-NPs) were examined by scanning electron microscopy (SEM) (Figure 1A,B), revealing well-defined particles with spherical to slightly faceted shapes and moderate surface roughness, features that may facilitate analyte access to the recognition sites. These structural characteristics are governed by the precipitation polymerization process carried out in acetonitrile, a solvent whose high polarity and low boiling point promote controlled phase separation and the formation of nanostructured porosity during particle growth [49,50]. Image analysis showed an average diameter of 135 ± 28 nm with a relatively narrow size distribution. Such a value is consistent with those commonly reported for MIPs prepared via precipitation polymerization with MAA and EGDMA in acetonitrile [49,51,52].

For comparison, limonene-imprinted nanoparticles prepared using the same molar ratio (1:4:8, LIN:MAA:EGDMA) exhibited a larger average diameter of about 179 ± 43 nm [37]. As reported in our previous study, non-imprinted nanoparticles (NIPs) synthesized under identical polymerization conditions exhibited an average diameter of 117 ± 28 nm. The slightly larger size observed for the linalool-imprinted MIPs suggests that the presence of the template molecule modulated the polymerization process and particle growth dynamics.

The reduction in particle size and improved uniformity observed for linalool-imprinted MIP-NPs likely stems from stronger template–monomer interactions. LIN, possessing a hydroxyl group, is expected to establish specific hydrogen bonds with MAA, resulting in stable pre-polymer complexes that direct nucleation and growth. Conversely, apolar limonene relies on weaker van der Waals forces, leading to less defined nucleation and broader particle size distribution.

Furthermore, the polarity of linalool increases its solubility in the polymerization medium, favouring a more uniform distribution of the template and promoting homogeneous nucleation. In contrast, limonene, being less polar, may disturb the solvent’s homogeneity, leading to fewer nucleation sites and the formation of larger particles. The use of ethylene glycol dimethacrylate (EGDMA) as a crosslinker ensures structural rigidity in all formulations. However, in the case of linalool, the combination of specific monomer–template interactions and sufficient crosslinking results in more compact and reproducible imprinted structures.

### 3.2. Nanofibrous Layer Characterization

**Figure 2:**
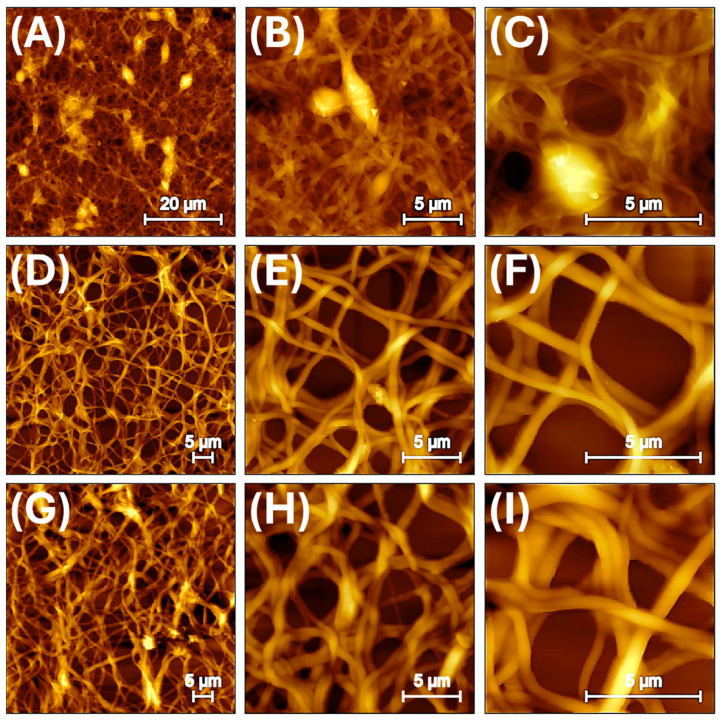
AFM topographic images (tapping mode, DynAl19 cantilever) of electrospun nanofibrous mats incorporating linalool-imprinted polymer nanoparticles (MIP-NPs) at increasing loadings: MIP_2.1_ (**A**–**C**), MIP_23_ (**D**–**F**), and MIP_40_ (**G**–**I**), corresponding to 2.1%, 23%, and 40% *w*/*w* MIP content relative to the polymer matrix, respectively. Images are shown at three different scan areas: 60 × 60 µm (**left**), 20 × 20 µm (**centre**), and 10 × 10 µm (**right**).

Atomic force microscopy (AFM) was employed to evaluate the morphological changes and surface topography of electrospun nanofibres as a function of increasing MIP nanoparticle loading. Nanofibres incorporating varying weight percentages of MIP nanoparticles synthesized with linalool as template were analysed to assess fibre diameter, surface roughness, and structural features such as bead formation. This analysis aims to elucidate the influence of MIP nanoparticle incorporation on the nanoscale architecture and surface properties of the fibres. At low MIP concentration (2.1%) (Figure 2A–C), the AFM images reveal a well-interconnected network of thin fibres forming a dense, fibrous mat. The samples are arranged vertically from top to bottom in order of increasing MIP content, while the horizontal arrangement from left to right corresponds to increasing magnification. A noticeable number of beads are present along the fibre axis, which is consistent with the effects of lower polymer solution viscosity as previously observed in electrospun PVP/MIP systems incorporating MIPs for limonene [37].

Topographical details of MIP_2.1_ (Figure 2A–C), derived from Gwyddion analysis following established methodologies for AFM surface characterization [53], reveal a fibrous surface morphology interspersed with globular protrusions. The root mean square roughness (Sq = 239.1 nm) and the average roughness (Sa = 176.6 nm) quantify substantial vertical deviations across the mat, consistent with the combined presence of nanoscale fibres and micrometric topographical features. The positive skewness (Ssk = 1.18) reflects an asymmetrical height distribution with a prevalence of elevated structures, while the sub-Gaussian kurtosis (Sku = 2.33) indicates a relatively broad distribution of surface heights. These values are coherent with the visual presence of rounded protrusions embedded within the electrospun network. Although local variations are clearly visible in the AFM micrographs, the grain-wise RMS roughness remains spatially uniform, suggesting a globally homogeneous texture with discrete, yet recurrent, morphological heterogeneities likely associated with the incorporation of MIP nanoparticles. At low MIP content, the polymer solution appears to maintain sufficient electrospinnability to generate continuous fibres. However, the reduced polymer density associated with lower MIP loading may enhance jet instability during electrospinning, potentially promoting the formation of beads. These findings are consistent with our previous study [37] using the same MIP content, in which SEM, TEM, and HR-TEM analyses confirmed the co-distribution of MIP-NPs and MWCNTs along the electrospun fibres, with a marked accumulation of nanoparticles within the bead regions.

With an intermediate MIP/PVP ratio of about 23%, the electrospun nanofibres display a more regular and uniform morphology, with a notable reduction in bead formation (Figure 2D–F). This morphological improvement is attributed to the increased mass loading and solid content of the polymer solution induced by the higher MIP loading, which likely promotes greater jet stability during electrospinning.

**Figure 3:**
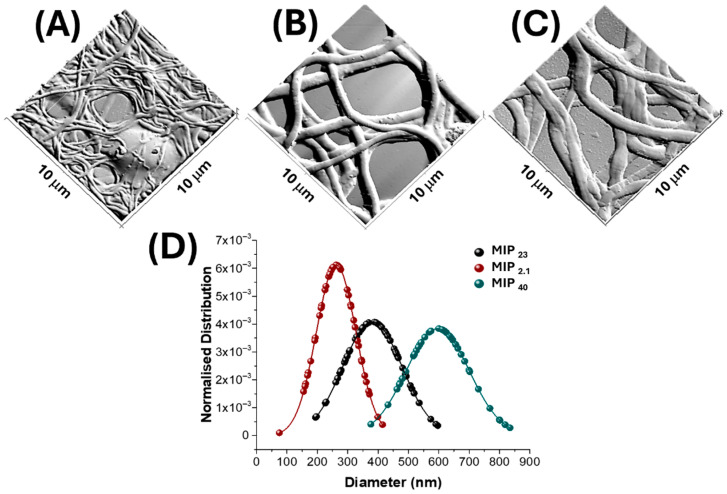
The figure shows 3D-AFM images (amplitude mode, 10 × 10 µm) of electrospun nanofibres containing increasing amounts of molecularly imprinted polymer nanoparticles (MIP-NPs): (**A**) MIP_2.1_, (**B**) MIP_23_, and (**C**) MIP_40_. (**D**) Fibre diameter distributions fitted with Gaussian functions based on 100 measurements per sample (MIP_2.1_ = 262 ± 65 nm (red), MIP_23_ = 381 ± 98 nm (black), MIP_40_ = 596 ± 104 nm (blue)).

Quantitative analysis reveals a significant increase in average fibre diameter, from ~262 nm to ~381 nm, accompanied by a broader yet still Gaussian size distribution (Figure 3D and Table 1). This is evidenced by the flattened and widened profile of the histogram, indicating a more polydisperse fibre population as the MIP content increases by nearly an order of magnitude compared to the lowest concentration.

Surface characterization by AFM shows a higher surface roughness, with an RMS roughness (Sq) of 339 nm and a mean roughness (Sa) of 282 nm. While these values remain within the typical range for electrospun nanofibres (from ~0.5 nm up to several hundreds of nanometres depending on the measurement area and method) [54], they indicate a moderate topographic complexity, likely associated with the incorporation of MIP domains along the fibre surface.

Interestingly, the height distribution appears statistically symmetric, with a skewness (Ssk) close to 0 and excess kurtosis near 0, suggesting a near-Gaussian distribution of surface heights, without pronounced protrusions or depressions. Moreover, the identical values for global and grain-wise RMS roughness (~339 nm) reflect a spatially homogeneous surface texture, supporting the notion of an even dispersion of MIP particles within the polymer matrix without inducing localized structural disruptions.

These findings indicate that increasing the MIP content to an intermediate level results in enhanced morphological regularity and topographic uniformity, making this composition particularly suitable for applications requiring controlled architectures.

At the highest MIP loading (Figure 2G–I), corresponding to 40% *w*/*w*, morphological alterations become more pronounced with fibres appearing thicker (average diameter ≈ 596 nm, Figure 3D and Table 1) and more tightly entangled, suggesting increased inter-fibre adhesion. AFM images clearly show the disappearance of discrete spherical protrusions seen at lower loadings, in favour of a more continuous, undulating surface texture. This transition is consistent with a more diffuse topographic irregularity, possibly arising from the partial coalescence or redistribution of MIP domains along the fibre surface.

Topographical data support these observations: the RMS roughness (Sq = 354 nm) and mean roughness (Sa = 276.6 nm) remain elevated, reflecting persistent height variability. However, the reduced skewness (Ssk = 0.6624) and moderate kurtosis (Sku = 1.152) indicate a smoother, less peaked height distribution compared to intermediate loadings. These changes suggest a shift from localized, protrusion-like heterogeneities to a more widespread but subtler surface roughness, likely induced by interfacial reorganization at high MIP concentrations. The morphological benefits associated with higher surface area and roughness may, at this stage, be offset by decreased control over fibre formation, potentially impacting sensor consistency and scalability.

Taken together, these results demonstrate that, although a higher MIP content can contribute to a more functionally rich and interactive surface, it requires careful optimization. Balancing imprinting efficiency with electrospinnability and morphological integrity is key. However, the fibres at this concentration remain structurally continuous and may still be advantageous for advanced sensing platforms, especially where enhanced surface–analyte interaction and high morphological complexity are desired.

The 3D topographical reconstructions presented in Figure 3A–C, derived from AFM amplitude images, highlight the impact of increasing MIP content on both fibre morphology and surface organization. Amplitude-mode imaging, being particularly sensitive to lateral force variations, enhances the contrast of surface discontinuities, such as granular domains or nanoparticle clusters.

At the lowest MIP content (2.1 wt%, Figure 3A), the fibres appear irregular and highly entangled, with thin diameters (Figure 3D) and a coarse surface texture. Numerous granular structures are clearly visible along the fibre surfaces, which can be correlated with partially exposed MIP nanoparticles, indicating a heterogeneous and non-uniform integration at this concentration.

At the intermediate MIP level (23 wt%, Figure 3B), the fibre morphology becomes more defined and regular, with improved alignment and reduced bead formation. Notably, the granular features are still present but appear more evenly dispersed, suggesting a better incorporation and stabilization of MIP-NPs within the polymer matrix. The surface texture remains complex but more coherent and continuous, likely reflecting improved compatibility between the MIP particles and the polymer solution at this concentration.

At the highest MIP loading (40 wt%, Figure 3C), the fibres exhibit larger diameters (Figure 3D) and a denser, more compact structure. Interestingly, granular features become less distinct, suggesting that the MIP-NPs may be embedded more deeply within the fibre matrix or covered by a thicker polymer layer. This could result from partial aggregation or saturation effects during electrospinning, which reduce the surface availability of the particles despite their higher bulk concentration.

**Figure 4:**
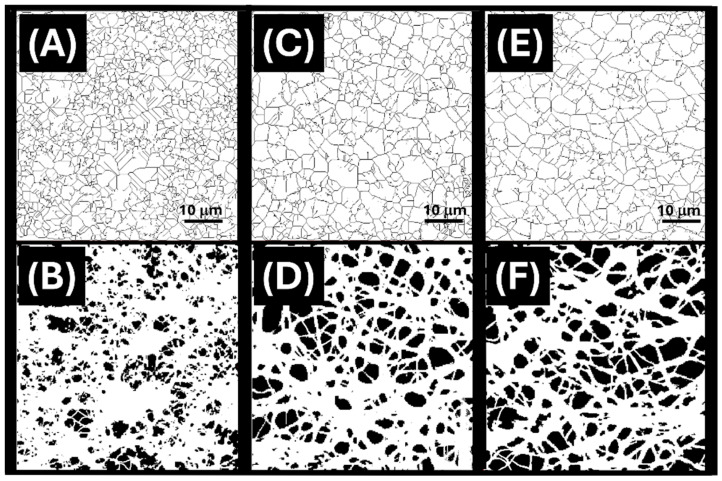
Binary image analysis of AFM topographic micrographs (60 × 60 µm) of electrospun nanofibres incorporating increasing concentrations of MIP-NPs: MIP_2.1_ (**A**,**B**), MIP_23_ (**C**,**D**), and MIP_40_ (**E**,**F**). Top panels (**A**,**C**,**E**): reprocessed AFM topography images obtained via ImageJ analysis highlighting the architecture of the fibrous network (plugin DiameterJ). Bottom panels (**B**,**D**,**F**): corresponding binary thresholded images used to quantify pore characteristics.

Furthermore, binary thresholding and particle analysis provided by Image J were applied to AFM micrographs (Figure 4A–F) to extract structural parameters including mean pore diameter, pore area, porosity percentage, and pore density.

For the lowest MIP loading (2.1%, Figure 4A,B), the nanofibrous network exhibited an average pore diameter of 3.2 ± 2.5 μm, with a mean pore area of 1.5 ± 3.2 μm^2^. The calculated percent porosity was approximately 22%, with a pore density of 0.14 pores/μm^2^. At an intermediate loading (23%, Figure 4C,D), the fibre mat showed a moderate increase in pore diameter and area, reaching 4.0 ± 3.0 μm and 2.9 ± 4.2 μm^2^, respectively. The corresponding porosity increased to 27%, although pore density decreased to 0.08 pores/μm^2^, suggesting the formation of fewer but larger pores.

For the highest MIP content (40%, Figure 4E,F), the analysis revealed a further increase in mean pore area to 4.3 ± 5.1 μm^2^, with pores ranging from 0.1 to 31.6 μm^2^ in size. The total porosity reached 36%, indicating a more open and interconnected structure. These changes are consistent with the hypothesis that higher MIP loading alters the fibre deposition dynamics and promotes the generation of larger inter-fibre voids.

A visual comparison of the AFM elaboration (Figure 4A,C,E) also suggests a progressive reduction in the number of visible fibres intersections with increasing MIP content.

At low MIP loading (2.1%), the nanofibrous mat (Figure 4A) appears densely entangled, with numerous crossing points contributing to smaller and more irregular pores (Figure 4B). In contrast, higher MIP loadings (23% and 40%) result in thicker, more loosely packed fibres, leading to a decrease in intersection density (Figure 4C,E) and the formation of larger, well-defined pore domains (Figure 4D,F). This morphological evolution is consistent with the observed increase in mean pore area and porosity and may also influence mechanical cohesion and analyte diffusion pathways within the mat.

The main morphological parameters of electrospun nanofibres with increasing MIP content are summarized in Table 1.

### 3.3. Electrical and Sensing Characterization

**Figure 5:**
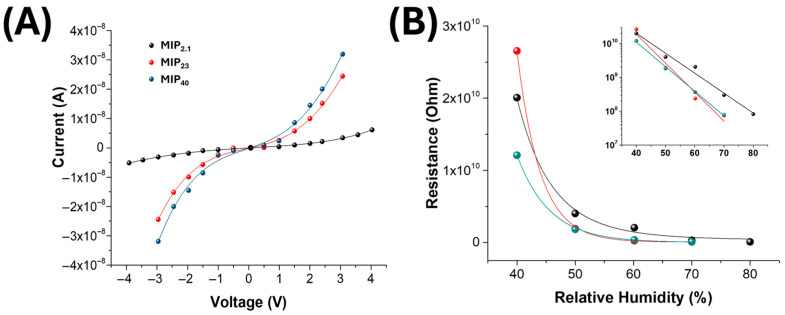
(**A**) I–V curves of electrospun nanofibre mats incorporating different amounts of MIP nanoparticles (MIP_2.1_—black, MIP_23_—red, MIP_40_—blue), measured under humid conditions (RH: 60%). The current response increases with MIP loading, indicating enhanced charge transport across the nanofibrous matrix due to the presence of conductive pathways facilitated by the MIP-NPs. (**B**) Variation in electrical resistance as a function of relative humidity (RH) for the same materials.

Figure 5A displays the current–voltage (I–V) characteristics of chemiresistive devices based on electrospun nanofibres loaded with increasing concentrations of linalool-imprinted MIP nanoparticles (2.1 wt%, 23 wt%, and 40 wt%, shown in black, red, and blue, respectively). The curves exhibit a diode-like response at low voltages and transition into a quasi-linear regime above ±1.5 V, allowing approximate resistance estimation using Ohm’s law.

A clear trend of enhanced electrical conductivity is observed with rising MIP content. This enhancement can be attributed to the combined effect of several factors. First, the hygroscopic nature of the MIP matrix enhances water uptake and promotes ionic conduction under ambient conditions, a phenomenon commonly observed in polymer-based humidity sensors [55,56,57]. Water molecules act as proton carriers or facilitate ionic transport through the dissociation of carboxyl or hydroxyl groups embedded in the polymer framework. A second factor may be the improved dispersion or alignment of carbon nanotubes (MWCNTs) within the fibrous matrix at higher MIP loading, thereby enhancing percolation pathways and electronic conduction [57,58]. It is well established that MWCNT arrangement within electrospun fibres is sensitive to polymer viscosity, jet stretching, and nanoparticle content [59]. The increasing MIP-NP concentration in the electrospun formulation may influence the overall viscoelasticity and charge density of the polymeric jet, which are key factors determining the stretching dynamics and internal organization of nanofillers such as multi-walled carbon nanotubes (MWCNTs). Higher MIP loading has been shown to alter fibre morphology and density and may consequently affect the spatial distribution or alignment of MWCNTs during fibre formation. Although direct experimental evidence was not obtained in this work, previous studies suggest that nanoparticle content can modulate nanotube orientation by changing the viscosity of the solution, the jet stability and elongational flow and the interfacial interactions between the dispersed phases [60,61]. It is therefore reasonable to hypothesize that the presence of MIP-NPs at increasing concentrations indirectly contributes to enhanced percolation or more favourable arrangements of MWCNTs, partly explaining the observed conductivity increase. Additionally, microstructural effects, such as increased fibre diameter or inter-fibre contact points at higher MIP concentrations, may reduce junction resistance and promote continuous charge transport.

These findings confirm that the incorporation of MIPs not only enables selective molecular recognition but also modulates the bulk electrical properties of the nanocomposite, with clear implications for device sensitivity and operational stability.

Figure 5B shows the effect of relative humidity (RH) on the baseline resistance of the electrospun sensing layers as a function of MIP loading. All the sensing layers exhibit an exponential decrease in resistance with increasing RH, which can be attributed to the water-induced enhancement of charge transport. As RH increases, water molecules are progressively adsorbed onto the polymeric surface and within the porous fibrous structure, forming a thin physisorbed water layer that facilitates ionic conduction through proton hopping (Grotthuss mechanism) and enhanced charge carrier mobility. It is worth noting that pristine PVP is intrinsically water-soluble; therefore, the UV crosslinking of the electrospun mats was performed to ensure structural integrity and prevent dissolution or morphological collapse under high humidity conditions. This stabilization step is essential for maintaining the reliability of chemiresistive measurements, particularly at elevated RH levels.

At low humidity (~40% RH), insufficient water adsorption results in high resistance, especially for the MIP_2.1_ sensor (R = 2.01 ± 0.10 × 10^10^ Ω), whereas MIP_23_ and MIP_40_ show slightly lower values (2.65 ± 0.13 × 10^10^ Ω and 1.21 ± 0.06 × 10^10^ Ω, respectively), indicating that higher MIP content improves hydrophilicity and initial conduction pathways.

Between 40% and 60% RH, resistance drops by 1–2 orders of magnitude due to the formation of continuous hydration layers that enable efficient ionic conduction. At 60% RH, the resistance converges within the 10^8^–10^9^ Ω range (e.g., R_(MIP23)_ = 2.37 ± 0.12 × 10^8^ Ω), reflecting the transition from dry-state conduction to water-assisted transport.

The inset of Figure 5B highlights the nearly linear relationship between log(R) and RH, emphasizing the systematic decrease and the saturation behaviour above ~50% RH. At higher humidity levels, additional water adsorption no longer increases conduction significantly, suggesting that the available conduction pathways are already saturated.

These observations confirm that RH is a key factor in modulating the electrical properties of the sensing layers, and that the MIP loading can be tuned to balance baseline resistance and humidity tolerance for optimized B-VOC detection under moisture-rich conditions.

**Figure 6:**
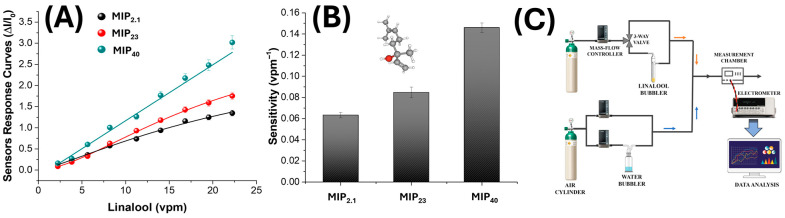
(**A**) Sensor response curves (ΔI/I_0_) of electrospun MIP-based nanofibres incorporating increasing MIP-NP loadings (MIP_2.1_, MIP_23_, and MIP_40_), measured under exposure to ascending linalool concentrations (2–22 vpm) at 60% relative humidity. (**B**) Sensitivity values defined as the slope of the linear response (ΔI/I_0_ vs. concentration) extracted from the linear fits in panel (**A**) and expressed in vpm^−1^. The 3D structure in the panel corresponds to a linalool molecule, the template used for imprinting. (**C**) A schematic diagram of the gas sensing measurement setup.

Figure 6A shows the calibration curves obtained for three sensors and performed at 60% RH upon exposure to progressively higher concentrations of linalool vapour (2–22 vpm). In all cases, the normalized response (ΔI/I_0_) increased with analyte concentration, confirming effective interaction between linalool and the MIP-based sensing layers. Notably, the sensor incorporating 40% MIP exhibited a clear linear response throughout the tested range (R^2^ = 0.991), indicating a large availability of accessible binding sites and a non-saturating regime within this concentration window. This enhanced response can be attributed to the structural evolution of the sensing layer with increasing MIP content. As reported in Table 1, the MIP_40_ nanofibrous mat exhibits a surface-textured network with increased roughness, which enhances the surface-to-volume ratio and the accessibility of the imprinted binding cavities. Compared to the 2.1% and 23% formulations, the MIP_40_ mat also displays a larger mean pore area and higher porosity. This combination of morphological and chemical factors results in a more open and permeable architecture, promoting efficient VOC diffusion and maximizing analyte–template interactions within the imprinted sites.

In contrast, the sensors with lower MIP content (2.1% and 23%) displayed non-linear behaviour, particularly at higher concentrations. The response curves showed a typical Langmuir-like trend, with an initial linear region followed by a sublinear increase, suggesting the partial saturation of the available recognition sites. This behaviour is consistent with a lower density of imprinted cavities and potential diffusion limitations in more compact nanofibrous networks.

The calculated sensitivity values, shown in Figure 6B, reflect these trends. Sensitivity was defined as the slope of the calibration curve in the linear region and expressed in vpm^−1^. A pronounced increase in sensitivity was observed as MIP content increased: from 0.064 vpm^−1^ for the 2.1% sensor to 0.099 vpm^−1^ at 23% and up to 0.146 vpm^−1^ for the 40% formulation. The improved sensitivity at higher MIP concentrations can be attributed to both a greater number of binding sites and improved accessibility within the more open and porous fibre network, as previously confirmed by AFM analysis.

These results collectively suggest that the functional performance of the chemiresistive sensor platform is tightly correlated with the amount of imprinted polymer embedded in the nanofibrous matrix. An optimal MIP concentration not only maximizes sensitivity, but also may ensure a linear and predictable response, which is critical for quantitative VOC detection in real-world applications.

**Figure 7:**
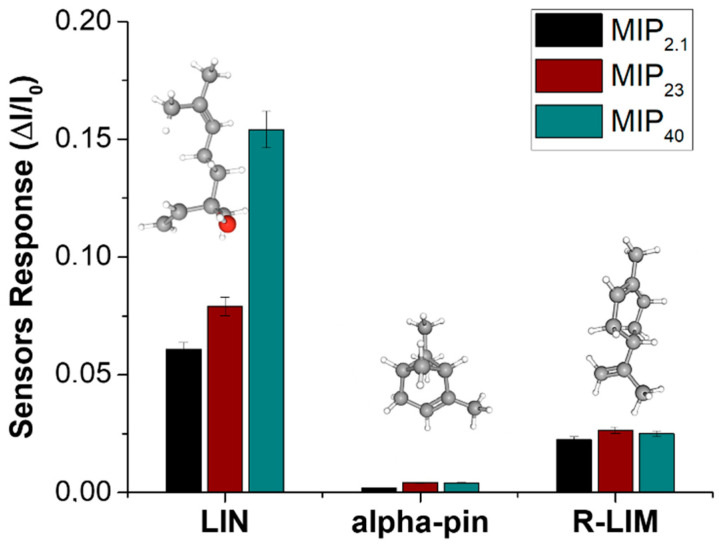
Bar plot of comparative sensor responses (ΔI/I_0_) of nanofibrous mats containing different MIP-NP loadings (MIP_2.1_, MIP_23_, and MIP_40_) exposed to 1 vpm of three different monoterpenes, linalool (LIN), α-pinene (alpha-pin), and R-(+)-limonene (R-LIM), all measured at 60% RH.

To evaluate the selectivity of the MIP-based chemiresistive sensors, their responses were compared under controlled conditions (VOC: ~1 vpm; RH: 60%) upon exposure to linalool (LIN) and two structurally related volatile monoterpenes: α-pinene (α-Pin) and R(+)-limonene (R-LIM). The results are shown in Figure 7. All three sensors exhibited a markedly higher response to linalool compared to the interferents, with selectivity clearly improving as the MIP content increased from 2.1% to 40%. For the lowest MIP-loaded sensor (MIP_2.1_), a moderate yet distinct preference for linalool was observed, while the selectivity improved for the 23% formulation and was most pronounced in the 40% MIP sensor, which showed a highly specific response pattern.

Quantitatively, the selectivity index (SI), defined as the ratio of the linalool response to the sum of the responses toward both interferents, was calculated as 71.35%, 72.05%, and 84.16% for the MIP_2.1_, MIP_23_, and MIP_40_ sensors, respectively. This progressive enhancement confirms that higher concentrations of MIP nanoparticles contribute to more efficient molecular imprinting, thereby increasing the density and definition of recognition cavities within the nanofibrous matrix. The minimal responses to α-pinene and R-limonene, despite their structural similarity to linalool (notably the shared monoterpene backbone), indicate that the sensors’ recognition mechanism is not governed merely by size or volatility, but by specific interactions, most likely hydrogen bonding, between the hydroxyl moiety of linalool and the carboxylic acid groups of the imprinted polymer. These results support the conclusion that MIP concentration plays a central role not only in boosting sensitivity, as shown previously, but also in refining the chemical specificity of the sensing platform, a critical requirement for the selective detection of volatile markers in complex environments.

**Figure 8:**
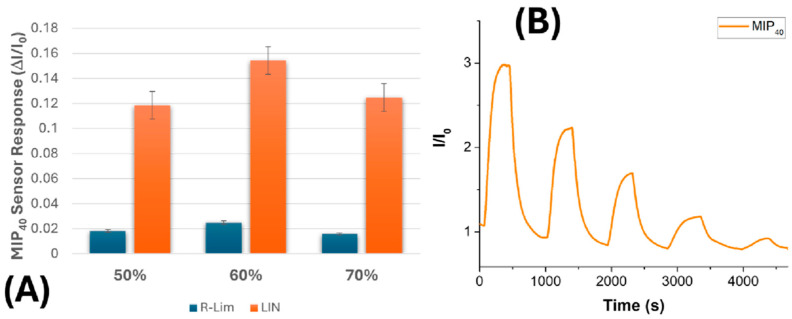
(**A**) The selective response of the MIP_40_ sensor to 1 vpm linalool (LIN, orange bar) and R-(+)-limonene (R-LIM, blue bar) at three different relative humidity levels (50%, 60%, and 70%). (**B**) The transient sensor response curve (I/I_0_) of the MIP_40_ formulation upon exposure to a descending concentration sequence of linalool vapour (22, 15, 10, 5, and 2 vpm) at 60% RH.

Figure 8A illustrates the normalized response of the MIP_40_ sensor to linalool (LIN, 1 vpm) and to the structural interferent R-(+)-limonene (R-LIM, 1 vpm) at three different levels of relative humidity (RH): 50%, 60%, and 70%. In all cases, the signal generated by linalool is consistently and significantly higher than that produced by R-limonene, confirming the sensor’s ability to maintain molecular selectivity under humid conditions.

Here, the SI increased from 76% at 50% RH, peaked at 89% at 60% RH, and then slightly decreased to 82% at 70% RH. This behaviour is consistent with previous observations where moderate humidity levels enhance polymer flexibility and analyte diffusion, leading to improved molecular recognition and signal amplification. Although the differences between these values are not extreme, the trend suggests that moderate humidity (around 60%) enhances sensor selectivity, likely by facilitating ion transport within the polymer matrix without overly disrupting the specific binding interactions. At 70% RH, the slight drop in selectivity can be explained by the increased water adsorption and partial swelling of the nanofibrous network, which may reduce the strength of specific template–monomer interactions or promote non-selective adsorption. Nevertheless, the response remains significantly higher for linalool compared to R-limonene, confirming the overall robustness of the MIP–template interactions.

This behaviour highlights the importance of controlling ambient humidity during sensor operation and suggests that 60% RH may represent an optimal balance between ionic conductivity and molecular recognition in this system. Previous studies suggest that ambient water can enhance the interaction between MIPs and VOCs by facilitating molecular diffusion, template rebinding, and hydrogen bond interactions [37,62]. Water can act as a plasticizer by increasing the mobility of polymer chains, thereby facilitating the rebinding of the template molecule within the recognition cavities of the MIP [63,64]. These effects may explain the slightly higher response of the MIP_40_ sensor at 60% RH compared to 50% RH, where lower hydration may limit the analyte accessibility to the active binding sites. This behaviour has been demonstrated in nanofibrous MIP-based sensors, where increased humidity enhances sensitivity by improving analyte access to binding sites [21,37]. Moreover, a recent review on MIP-based VOC sensors highlights that water adsorption within the polymer matrix can enhance the accessibility of recognition sites, thereby increasing effective analyte binding [22].

These results support the hypothesis that moderate levels of ambient humidity may enhance the selective rebinding of linalool to its imprinting sites by facilitating hydrogen bonding networks mediated by water molecules. The resulting increase in signal intensity at 60% RH therefore represents an optimal trade-off between water-mediated conduction and molecular recognition efficiency. This mechanism may contribute to improved sensitivity and performance of chemiresistive sensors for biogenic VOCs.

Figure 8B shows the normalized current response (I/I_0_) of the MIP_40_-based sensor upon exposure to a descending concentration sequence of linalool vapour (approximately 22, 15, 10, 5, and 2 vpm) at 60% relative humidity. Each exposure pulse results in a distinct increase in the normalized signal, followed by a complete return to baseline upon the reintroduction of clean air, indicating negligible sensor drift and low noise. The baseline signal remained stable over time, supporting the sensor’s robustness and reversibility under humid conditions. The response time (t_90_), defined as the time required to reach 90% of the maximum signal, was estimated to be ≤35 s for the first two transients, while the recovery time (t_10_), defined as the time required for the sensor signal to return to 10% above its baseline value after the removal of the target analyte, was <60 s. The progressive decrease in peak amplitude mirrors the descending linalool concentrations, confirming good signal proportionality in the low vpm range. Importantly, the presence of ambient moisture (60% RH) does not hinder the sensor response; rather, it appears to facilitate ion-mediated charge transport through the imprinted nanofibrous matrix.

Based on the standard 3σ criterion (three times the baseline noise), the limit of detection (LOD) of the MIP_40_ sensor was calculated to be approximately 8 ppb (±1), underscoring the notable sensitivity of the proposed nanostructured architecture [65,66]. To place these findings within the context of recent advances in linalool sensing, a comparative analysis is provided, focusing on selected examples drawn from the literature (Table 2). These include quartz crystal microbalance (QCM) sensors, metal oxide semiconductors (MOSs), electrochemical and colorimetric systems, photoionization detectors (PIDs), and multi-sensor arrays (e-noses), which represent some of the most relevant strategies for comparison. QCM sensors, particularly those functionalized with PEG [67] or hybrid materials such as HKUST-1/MWCNTs/aerogel@MIP [68], are among the most sensitive reported for linalool, reaching LODs of about 200 ppb under dry conditions. While effective in controlled environments, their performance in humid conditions remains largely unexplored, and their mechanical or thermal stability may be less robust than that of electrospun polymer-based systems.

MOS-based sensors, including those based on WO_3_, WS_2_, and WO_3_–WS_2_ nanocomposites, offer relatively simple fabrication and detection limits in the 107–270 ppb range [69]. Similarly, CuO nanoflake-based resistive sensors, which benefit from a high surface area and efficient charge transport, have achieved LODs as low as 28 ppb [70]. However, MOS sensors generally lack molecular specificity and require elevated operating temperatures (200–300 °C), which compromises energy efficiency and limits portability, factors that are critical for applications in open or wearable systems.

Electrochemical sensors, such as screen-printed carbon electrodes (SPCEs), offer good sensitivity (~12 vpm in liquid phase) but are mostly suited for solution-phase detection and lack direct gas-phase applicability [71].

Colorimetric or biomimetic sensors provide qualitative detection based on catalytic or redox reactions but are rarely quantitative or robust for continuous monitoring [72].

Electronic noses, combining multiple semi-selective sensors, and PID devices, designed for broad-spectrum VOC detection, lack the compound specificity required to selectively detect linalool without algorithmic post-processing [73].

The results obtained with the MIP_40_ sensor underscore a combination of performance features that distinguish this approach from other state-of-the-art systems. The sensor is capable of achieving ppb-level detection under ambient conditions, including room temperature and high relative humidity (60% RH), while maintaining rapid response times and full signal reversibility. These characteristics stem from the synergistic integration of molecularly imprinted nanoparticles into an electrospun nanofibre matrix, which supports efficient analyte diffusion, selective molecular recognition, and ion-mediated charge transport.

Furthermore, this sensing strategy offers a level of tunability rarely achievable with monolithic or single-material devices. By adjusting the MIP loading within the fibrous scaffold, the sensor’s sensitivity, selectivity, and linear dynamic range could be tailored to specific detection requirements, without the need for modifying the sensing chemistry or fabrication protocol. This architectural flexibility not only broadens the range of potential applications, from environmental and agricultural VOC monitoring to real-time sensing in complex gas matrices, but also provides a robust framework for future optimization and functional diversification.

**Figure 9:**
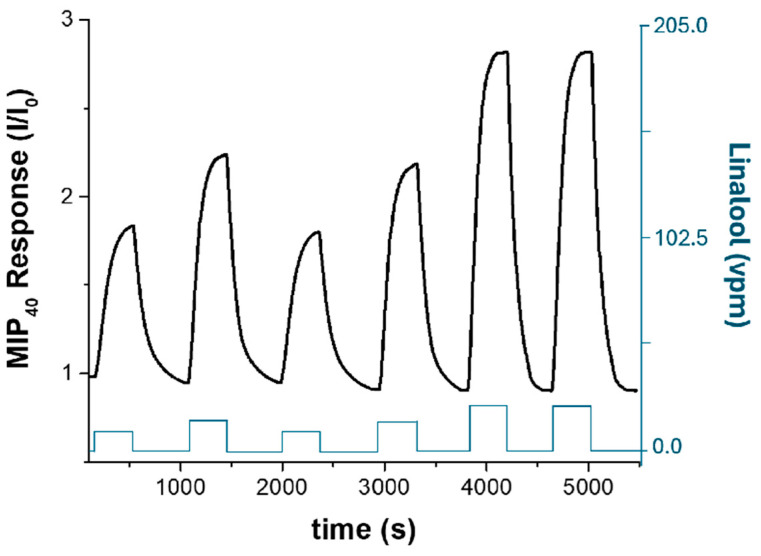
Transient response of the MIP_40_ sensor to successive linalool vapour pulses (60% RH). The black curve (left axis) represents the normalized current response (I/I_0_), while the blue stepped curve (right axis) indicates the applied linalool vapour concentrations (vpm), following the sequence 10, 15, 10, 15, 22, and 22 vpm.

To verify the reproducibility of the sensor response, replicate measurements were carried out under stabilized conditions, as shown in Figure 9. The transient profiles and peak amplitudes obtained for repeated exposures to the same linalool concentrations exhibit minimal variation (<5%), confirming that the sensing layer provides consistent and reliable responses. The baseline drift observed in Figure 8 (about −0.072 h^−1^) may be attributable to the initial transient stabilization phase immediately after system start-up and does not affect the accuracy of the recorded responses.

## 4. Conclusions

This study demonstrates a versatile and scalable sensor architecture for the selective detection of linalool, a representative biogenic volatile organic compound (B-VOC), based on the synergistic combination of molecular imprinting and electrospinning technologies. A key advantage of this system lies in its tunability, as sensor performance can be precisely modulated by adjusting the ratio between molecularly imprinted polymer nanoparticles (MIP-NPs) and the host polymer matrix. This design flexibility enables the rational engineering of sensing layers with optimized sensitivity, selectivity, and dynamic range. Linalool-imprinted MIP-NPs were synthesized via simple template-assisted precipitation polymerization using methacrylic acid (MAA) and ethylene glycol dimethacrylate (EGDMA), yielding uniform nanoparticles (~135 nm) without requiring complex purification steps. Their incorporation into polyvinylpyrrolidone (PVP) matrices, doped with multi-walled carbon nanotubes (MWCNTs), was carried out via electrospinning, a cost-effective and scalable technique that produces high-surface-area nanofibrous films with controllable morphology and porosity.

Morphological analyses showed that increasing MIP content influences fibre roughness and network architecture, while electrical tests under different humidity conditions revealed improved sensor performance at 60% RH, likely due to enhanced ion-mediated charge transport. The highest MIP loading (40%) provided the best results, with a detection limit of 8 ± 1 ppb and 84% selectivity over structurally related terpenes (α-pinene and R-(+)-limonene). In addition, the sensor response was shown to be reproducible, as confirmed by replicated measurements under controlled conditions. The sensors maintained consistent performance over a period of nearly two years from fabrication to final testing, suggesting robust long-term stability, which will be systematically addressed in future work.

Beyond performance, the combined use of imprinting and electrospinning offers advantages in terms of synthesis simplicity, compositional control, and scalability. Both the fabrication of MIP-NPs and the formation of nanofibrous mats are compatible with solvent-based processing, ambient conditions, and flexible substrates, facilitating integration into wearable or portable devices. Looking forward, this hybrid material system can be adapted to detect a wider range of volatile biomarkers by tailoring the molecular imprinting toward different targets. Its compatibility with low-power electronics and potential for multiplexed detection make it particularly suitable for environmental sensing, smart agriculture, and plant-emitted VOC monitoring.

Future work will address sensor stability, regeneration, and the optimization of selectivity and performance through alternative monomers and crosslinkers while investigating how increasing MIP-NP content affects the arrangement, dispersion, and connectivity of MWCNTs, critical factors for maintaining efficient conductive pathways.

Overall, the accessibility and adaptability of this approach support its advancement toward next-generation selective gas sensors.

## Figures and Tables

**Table 1:** Structural parameters of electrospun nanofibre mats as a function of MIP loading.

MIP Loading (%)	Fibre Diameter (nm)	Mean Pore Area (µm^2^)	Percent Porosity (%)
**2.1**	262 ± 65	1.5 ± 3.2	22
**23**	381 ± 98	2.9 ± 4.2	27
**40**	596 ± 104	4.29± 5.1	36

**Table 2:** Key features of recently reported linalool sensors.

Sensor Type	LOD	Operating Temp	Response Time	Selectivity	Ref.
**MIP-nanofibre chemiresistor**	~8 ppb (±1)	RT (60% RH)	<60 s (t_90_)	SI ~73% (vs R-limonene)	This work
**QCM (PEG-coated)**	~200 ppb	RT (dry)	~minutes	Limited VOC selectivity	[67]
**QCM (HKUST-1/MWCNT@MIP)**	~200 ppb	RT	~10 min exposure	High template specificity	[68]
**MOS (WO_3_, WS_2_, WO_3_–WS_2_)**	107–270 ppb	200–300 °C	Seconds (typical)	Non-specific to linalool	[69]
**CuO nanoflake resistive sensor**	~28 ppb	200–300 °C	Seconds (typical)	Low molecular specificity	[70]
**Electrochemical (SPCE)**	~12 vpm (liquid)	RT (liquid phase)	Seconds–minutes	Not applicable to gas-phase	[71]
**Colorimetric sensors**	(qualitative)	RT	Minutes	Qualitative, non-specific	[72]
**E-nose (sensor array)**	~ppm (broad VOCs)	RT	Seconds–minutes	Requires algorithmic processing	[73]

## Data Availability

The data presented in this study are available on request from the corresponding author. The data are currently stored in IRIS, the institutional repository of the National Research Council of Italy (CNR).

## References

[1] Luo R., Lun X., Gao R., Wang L., Yang Y., Su X., Habibullah-Al-Mamun M., Xu X., Li H., Li J. (2025). A Review of Biogenic Volatile Organic Compounds from Plants: Research Progress and Future Prospects. Toxics.

[2] Maffei M.E. (2010). Sites of Synthesis, Biochemistry and Functional Role of Plant Volatiles. South Afr. J. Bot..

[3] Erasto P., Viljoen A.M. (2008). Limonene-A Review: Biosynthetic, Ecological and Pharmacological Relevance. Nat. Prod. Commun..

[4] Qiu C.L., Li W., Wang L.N., Wang S.C., Falert S., Wang C., Yu S.Y., Abdelkhalek S.T., Lu J., Lin Y.J. (2025). Limonene Enhances Rice Plant Resistance to a Piercing-Sucking Herbivore and Rice Pathogens. Plant Biotechnol. J..

[5] Hosseini R., Heidari M. (2025). Impact of Drought Stress on Biochemical and Molecular Responses in Lavender (*Lavandula angustifolia* Mill.): Effects on Essential Oil Composition and Antibacterial Activity. Front. Plant Sci..

[6] de Lima Silva J.R., dos Santos L.B., Hassan W., Kamdem J.P., Duarte A.E., Soufan W., El Sabagh A., Ibrahim M. (2024). Exploring the Therapeutic Potential of the Oxygenated Monoterpene Linalool in Alleviating Saline Stress Effects on *Allium Cepa* L.. Environ. Sci. Pollut. Res..

[7] Ilc T., Parage C., Boachon B., Navrot N., Werck-Reichhart D. (2016). Monoterpenol Oxidative Metabolism: Role in Plant Adaptation and Potential Applications. Front. Plant Sci..

[8] Yue R., Li Y., Qi Y., Liang X., Zheng Z., Ye Z., Tong W., Si X., Zhang Y., Xia E. (2025). Divergent MYB Paralogs Determine Spatial Distribution of Linalool Mediated by JA and DNA Demethylation Participating in Aroma Formation and Cold Tolerance of Tea Plants. Plant Biotechnol. J..

[9] Lahmar I., Yotova L., Belghith K. (2025). Environmental Impact on Phonological Stages in *Lavandula officinalis*: Chemical Profiling of Essential Oil and Extract, Antioxidant Activity, and Acetylcholinesterase Inhibition Potential. Euro-Mediterr. J. Environ. Integr..

[10] Eghlima G., Saeed-Abadi B., Sonboli A., Rezadoost H., Mirjalili M.H. (2025). Phenotypic Yield-Attributed Traits, Essential Oil Content and Composition of Iranian *Grammosciadium platycarpum* (Apiaceae) Populations: A Rich Source of (S)-(+)-Linalool. BMC Plant Biol..

[11] Jiao C., Gong J., Guo Z., Li S., Zuo Y., Shen Y. (2022). Linalool Activates Oxidative and Calcium Burst and CAM3-ACA8 Participates in Calcium Recovery in Arabidopsis Leaves. Int. J. Mol. Sci..

[12] Wang K., Ren W., Hong L., Wang Q., Ghimire R., Haapanen M., Kivimäenpää M., Wu P., Ma X., Asiegbu F.O. (2025). Linalool and 1,8-Cineole as Constitutive Disease-Resistant Factors of Norway Spruce Against Necrotrophic Pathogen *Heterobasidion parviporum*. Plant Cell Environ..

[13] Ramanpong J., Tsao C., Yin J., Wu C.D., Huang Y.C., Yu C.P. (2025). Effects of Forest Bathing and the Influence of Exposure Levels on Cognitive Health in the Elderly: Evidence from a Suburban Forest Recreation Area. Urban For. Urban Green.

[14] Haluza D., Kersten P., Lazic T., Steinparzer M., Godbold D. (2025). Unlocking the Power of Nature: Insights from a 20-Minute Forest Visit on Well-Being. Forests.

[15] Srivastava D., Vu T.V., Tong S., Shi Z., Harrison R.M. (2022). Formation of Secondary Organic Aerosols from Anthropogenic Precursors in Laboratory Studies. NPJ Clim. Atmos. Sci..

[16] Li M., Cappellin L., Xu J., Biasioli F., Varotto C. (2020). High-Throughput Screening for in Planta Characterization of VOC Biosynthetic Genes by PTR-ToF-MS. J. Plant Res..

[17] Cappellin L., Loreto F., Aprea E., Romano A., Sánchez del Pulgar J., Gasperi F., Biasioli F. (2013). PTR-MS in Italy: A Multipurpose Sensor with Applications in Environmental, Agri-Food and Health Science. Sensors.

[18] Wasilewski T., Orbay S., Brito N.F., Sikora K., Melo A.C.A., Melendez M.E., Szulczyński B., Sanyal A., Kamysz W., Gębicki J. (2024). Molecularly Imprinted Polymers for the Detection of Volatile Biomarkers. TrAC Trends Anal. Chem..

[19] Leibl N., Haupt K., Gonzato C., Duma L. (2021). Molecularly Imprinted Polymers for Chemical Sensing: A Tutorial Review. Chemosensors.

[20] Hua Y., Ahmadi Y., Kim K.-H. (2022). Molecularly Imprinted Polymers for Sensing Gaseous Volatile Organic Compounds: Opportunities and Challenges. Environ. Pollut..

[21] Macagnano A., Molinari F.N., Papa P., Mancini T., Lupi S., D’Arco A., Taddei A.R., Serrecchia S., De Cesare F. (2024). Nanofibrous Conductive Sensor for Limonene: One-Step Synthesis via Electrospinning and Molecular Imprinting. Nanomaterials.

[22] Cowen T., Cheffena M. (2022). Template Imprinting Versus Porogen Imprinting of Small Molecules: A Review of Molecularly Imprinted Polymers in Gas Sensing. Int. J. Mol. Sci..

[23] Caldara M., van Wissen G., Cleij T.J., Diliën H., van Grinsven B., Eersels K., Lowdon J.W. (2023). Deposition Methods for the Integration of Molecularly Imprinted Polymers (MIPs) in Sensor Applications. Adv. Sens. Res..

[24] Gavrilă A.M., Stoica E.B., Iordache T.V., Sârbu A. (2022). Modern and Dedicated Methods for Producing Molecularly Imprinted Polymer Layers in Sensing Applications. Appl. Sci..

[25] Lowdon J.W., Diliën H., Singla P., Peeters M., Cleij T.J., van Grinsven B., Eersels K. (2020). MIPs for Commercial Application in Low-Cost Sensors and Assays—An Overview of the Current Status Quo. Sens. Actuators B Chem..

[26] Malitesta C., Mazzotta E., Picca R.A., Poma A., Chianella I., Piletsky S.A. (2012). MIP Sensors—The Electrochemical Approach. Anal. Bioanal. Chem..

[27] González-Vila Á., Debliquy M., Lahem D., Zhang C., Mégret P., Caucheteur C. (2017). Molecularly Imprinted Electropolymerization on a Metal-Coated Optical Fiber for Gas Sensing Applications. Sens. Actuators B Chem..

[28] Emam S., Adedoyin A., Geng X., Zaeimbashi M., Adams J., Ekenseair A., Podlaha-Murphy E., Sun N.X. (2018). A Molecularly Imprinted Electrochemical Gas Sensor to Sense Butylated Hydroxytoluene in Air. J. Sens..

[29] Debliquy M., Dony N., Lahem D., Tang X., Zhang C., Raskin J.P., Olivier M.G. (2016). Acetaldehyde Chemical Sensor Based on Molecularly Imprinted Polypyrrole. Procedia Eng..

[30] Ye X., Ge L., Jiang T., Guo H., Chen B., Liu C., Hayashi K. (2022). Fully Inkjet-Printed Chemiresistive Sensor Array Based on Molecularly Imprinted Sol-Gel Active Materials. ACS Sens..

[31] Jin M., Shi P., Sun Z., Zhao N., Shi M., Wu M., Ye C., Lin C.T., Fu L. (2024). Advancements in Polymer-Assisted Layer-by-Layer Fabrication of Wearable Sensors for Health Monitoring. Sensors.

[32] Dong C., Shi H., Han Y., Yang Y., Wang R., Men J. (2021). Molecularly Imprinted Polymers by the Surface Imprinting Technique. Eur. Polym. J..

[33] Azhdary P., Janfaza S., Fardindoost S., Tasnim N., Hoorfar M. (2023). Highly Selective Molecularly Imprinted Polymer Nanoparticles (MIP NPs)-Based Microfluidic Gas Sensor for Tetrahydrocannabinol (THC) Detection. Anal. Chim. Acta.

[34] Zhang J., Zhang X., Zhang Y., Yang X., Guo L., Man C., Jiang Y., Zhang W., Zhang X. (2025). Emerging Biosensors Integrated with Microfluidic Devices: A Promising Analytical Tool for on-Site Detection of Mycotoxins. NPJ Sci. Food.

[35] Kamyab H., Chelliapan S., Tavakkoli O., Mesbah M., Bhutto J.K., Khademi T., Kirpichnikova I., Ahmad A., ALJohani A.A. (2022). A Review on Carbon-Based Molecularly-Imprinted Polymers (CBMIP) for Detection of Hazardous Pollutants in Aqueous Solutions. Chemosphere.

[36] Macagnano A., Zampetti E., Kny E. (2015). Electrospinning for High Performance Sensors.

[37] Molinari F.N., Marelli M., Berretti E., Serrecchia S., Coppola R.E., De Cesare F., Macagnano A. (2025). Cutting-Edge Sensor Design: MIP Nanoparticle-Functionalized Nanofibers for Gas-Phase Detection of Limonene in Predictive Agriculture. Polymers.

[38] Wackerlig J., Lieberzeit P.A. (2015). Molecularly Imprinted Polymer Nanoparticles in Chemical Sensing—Synthesis, Characterisation and Application. Sens. Actuators B Chem..

[39] Nagalingam S., Seco R., Kim S., Guenther A. (2023). Heat Stress Strongly Induces Monoterpene Emissions in Some Plants with Specialized Terpenoid Storage Structures. Agric. For. Meteorol..

[40] Kask K., Kännaste A., Talts E., Copolovici L., Niinemets Ü. (2016). How Specialized Volatiles Respond to Chronic and Short-Term Physiological and Shock Heat Stress in Brassica Nigra. Plant Cell Environ..

[41] Fürstenberg-Hägg J., Zagrobelny M., Bak S. (2013). Plant Defense against Insect Herbivores. Int. J. Mol. Sci..

[42] Chacón-Fuentes M., Bardehle L., Seguel I., Espinoza J., Lizama M., Quiroz A. (2023). Herbivory Damage Increased VOCs in Wild Relatives of Murtilla Plants Compared to Their First Offspring. Metabolites.

[43] Stringari G., Villanueva J., Appolloni E., Orsini F., Villalba G., Gabarrell Durany X. (2024). Measuring BVOC Emissions Released by Tomato Plants Grown in a Soilless Integrated Rooftop Greenhouse. Heliyon.

[44] Boachon B., Junker R.R., Miesch L., Bassard J.E., Höfer R., Caillieaudeaux R., Seidel D.E., Lesot A., Heinrich C., Ginglinger J.F. (2015). CYP76C1 (Cytochrome P450)-Mediated Linalool Metabolism and the Formation of Volatile and Soluble Linalool Oxides in Arabidopsis Flowers: A Strategy for Defense against Floral Antagonists. Plant Cell.

[45] Avesani S., Lazazzara V., Robatscher P., Oberhuber M., Perazzolli M. (2023). Volatile Linalool Activates Grapevine Resistance against Downy Mildew with Changes in the Leaf Metabolome. Curr. Plant Biol..

[46] McCallum E.J., Cunningham J.P., Lücker J., Zalucki M.P., De Voss J.J., Botella J.R. (2011). Increased Plant Volatile Production Affects Oviposition, but Not Larval Development, in the Moth Helicoverpa Armigera. J. Exp. Biol..

[47] Molinari F., Medrano A.V., Bacigalupe A., Escobar M.M., Monsalve L.N. (2018). Different Dispersion States of MWCNT in Aligned Conductive Electrospun PCL/MWCNT Composites. Fuller. Nanotub. Carbon Nanostructures.

[48] Maciejewska B.M., Wychowaniec J.K., Woźniak-Budych M., Popenda Ł., Warowicka A., Golba K., Litowczenko J., Fojud Z., Wereszczyńska B., Jurga S. (2019). UV Cross-Linked Polyvinylpyrrolidone Electrospun Fibres as Antibacterial Surfaces. Sci. Technol. Adv. Mater..

[49] Djunaidi M.C., Putri V.R., Maharani N.D., Lusiana R.A., Siahaan P., Sunarno S. (2024). Precipitation Polymerization-Based Molecularly Imprinted Polymers: A Novel Approach for Transdermal Curcumin Delivery. Polymers.

[50] Holdsworth C.I., Lim K.F., Muang-Non P., Martín-Esteban A. (2021). Molecularly Imprinted Polymeric Nanoparticles by Precipitation Polymerization and Characterization by Quantitative NMR Spectroscopy. Molecularly Imprinted Polymers. Methods in Molecular Biology.

[51] Karnka R., Chaiyasat P., Chaiyasat A. (2017). Synthesis of Uniform and Stable Molecularly Imprinted Polymer Particles by Precipitation Polymerization. Orient. J. Chem..

[52] Hasanah A.N., Safitri N., Zulfa A., Neli N., Rahayu D. (2021). Factors Affecting Preparation of Molecularly Imprinted Polymer and Methods on Finding Template-Monomer Interaction as the Key of Selective Properties of the Materials. Molecules.

[53] Aglikov A.S., Zhukov M.V., Aliev T.A., Kozodaev D.A., Nosonovsky M., Skorb E.V. (2024). New Metrics for Describing Atomic Force Microscopy Data of Nanostructured Surfaces through Topological Data Analysis. Appl. Surf. Sci..

[54] Langwald S.V., Ehrmann A., Sabantina L. (2023). Measuring Physical Properties of Electrospun Nanofiber Mats for Different Biomedical Applications. Membranes.

[55] Raza S., Li X., Soyekwo F., Liao D., Xiang Y., Liu C. (2021). A Comprehensive Overview of Common Conducting Polymer-Based Nanocomposites; Recent Advances in Design and Applications. Eur. Polym. J..

[56] Alam N., Abid N., Islam S.S. (2024). Advancements in Trace and Low Humidity Sensors Technologies Using Nanomaterials: A Review. ACS Appl. Nano Mater..

[57] Alam M.W., Bhat S.I., Al Qahtani H.S., Aamir M., Amin M.N., Farhan M., Aldabal S., Khan M.S., Jeelani I., Nawaz A. (2022). Recent Progress, Challenges, and Trends in Polymer-Based Sensors: A Review. Polymers.

[58] Sundaray B., Subramanian V., Natarajan T.S., Krishnamurthy K. (2006). Electrical Conductivity of a Single Electrospun Fiber of Poly(Methyl Methacrylate) and Multiwalled Carbon Nanotube Nanocomposite. Appl. Phys. Lett..

[59] Yin Z., Ding A., Zhang H., Zhang W. (2022). The Relevant Approaches for Aligning Carbon Nanotubes. Micromachines.

[60] Ahmadi Bonakdar M., Rodrigue D. (2024). Electrospinning: Processes, Structures, and Materials. Macromol.

[61] Hossain M.M., Islam M.A., Shima H., Hasan M., Lee M. (2017). Alignment of Carbon Nanotubes in Carbon Nanotube Fibers Through Nanoparticles: A Route for Controlling Mechanical and Electrical Properties. ACS Appl. Mater. Interfaces.

[62] Feldner A., Völkle J., Lieberzeit P., Fruhmann P. (2023). Conductive Molecularly Imprinted Polymers (CMIPs): Rising and Versatile Key Elements in Chemical Sensing. Chemosensors.

[63] Zhang H. (2014). Water-Compatible Molecularly Imprinted Polymers: Promising Synthetic Substitutes for Biological Receptors. Polymer.

[64] Horemans F., Weustenraed A., Spivak D., Cleij T.J. (2012). Towards Water Compatible MIPs for Sensing in Aqueous Media. Mol. Recognit..

[65] Vial J., Jardy A. (1999). Experimental Comparison of the Different Approaches to Estimate LOD and LOQ of an HPLC Method. Anal. Chem..

[66] Taleuzzaman M. (2018). Limit of Blank (LOB), Limit of Detection (LOD), and Limit of Quantification (LOQ). Org. Med. Chem. Int. J..

[67] Sharma P., Ghosh A., Tudu B., Bhuyan L.P., Tamuly P., Bhattacharyya N., Bandyopadhyay R., Chatterjee A. (2014). Detection of Linalool in Black Tea Using a Quartz Crystal Microbalance Sensor. Sens. Actuators B Chem..

[68] Liu S., Wu X., Chen S., Liu C., Zhang W., Yang M., Cheng J., Lu G., Wang Z., Chen W. (2025). Rapid Detection of Linalool by QCM Gas Sensor Based on HKUST-1/MWCNT-Gel@MIP in Early Sweetpotato Black Spot Disease. Microchem. J..

[69] Mushannavar S., Ghosh R. (2025). Selective Detection of Linalool Using WO3-WS2 Composite Based Resistive Sensors. Sens. Actuators B Chem..

[70] Kulkarni S., Kummara S., Gorthala G., Ghosh R. (2022). CuO Nanoflake-Based Sensors for Detecting Linalool, Hexanal, and Methyl Salicylate. ACS Agric. Sci. Technol..

[71] Bhuyan A., Tudu B., Bandyopadhyay R., Gogoi S., Singh A. (2019). Preanodized Screen Printed Carbon Electrode for Detection of Linalool Using Three Terminal Network. Int. J. Eng. Adv. Technol..

[72] Li M., Dong S., Cao S., Cui Q., Chen Q., Ning J., Li L. (2023). A Rapid Aroma Quantification Method: Colorimetric Sensor-Coupled Multidimensional Spectroscopy Applied to Black Tea Aroma. Talanta.

[73] Zhou C., Fan J., Tan R., Peng Q., Cai J., Zhang W. (2022). Prediction of Linalool Content in Osmanthus Fragrans Using E-Nose Technology. J. Sens..

